# Supplementary material for: How does date-rounding affect phylodynamic inference for public health?
Source: PLoS Comput Biol. 2025 Apr 11;21(4):e1012900. doi: 10.1371/journal.pcbi.1012900 (PMC11991728; doi:10.1371/journal.pcbi.1012900)
Supplement: S1 File — (PDF) Fig A in S1 File. Mean posterior variance for parameters of interest for each replicate of simulated data varying across date resolution. Individual lines track mean posterior variance for each simulated dataset and boxplots are given to summarise variance in each condition at each date resolution. Rows correspond to individual parameters, columns correspond to simulation conditions (underlying parameters matching each empirical dataset), and colour corresponds to tree prior or reproductive number interval. (A) Mean posterior variance in substitution rate across simulation scenarios. (B) Posterior variance in tMRCA, a measure of the age of the population driving the outbreak. (C) Posterior variance in reproductive number reproductive number. (PDF) Fig B in S1 File. Desnsitrees (overlaid posterior trees) for empirical data with columns corresponding to pathogen under each combination of date resolution and tree prior. For the H1N1 and SARS-CoV-2 treatments, Year resolution causes trees to collapse to instantaneous bursts. (PDF) Fig C in S1 File. Adjusted phylodynamic likelihood against adjusted phylogenetic likelihood with panels corresponding to each simulation condition. Points correspond to mean posterior likelihood for each simulated dataset under each simulation condition. Colour corresponds to date resolution. Likelihoods are adjusted by subtracting the mean phylodynamic or phylogenetic likelihood at day resolution from each the means under month and year resolution. Resulting points therefore show the difference phylodynamic and phylogenetic likelihoods due to date-rounding with the point ( 0 , 0 ) representing likelihood at day resolution for each dataset. Month resolution generally results in smaller differences that Year resolution, suggesting coarser date resolution results in more perturbed likelihoods. There is also generally more error in phylodynamic likelihood than phylogenetic likelihood. (PDF) Fig D in S1 File. Posterior distributions for the [file pcbi.1012900.s001.pdf]

S1 Appendix

March 2, 2025

Fig. A

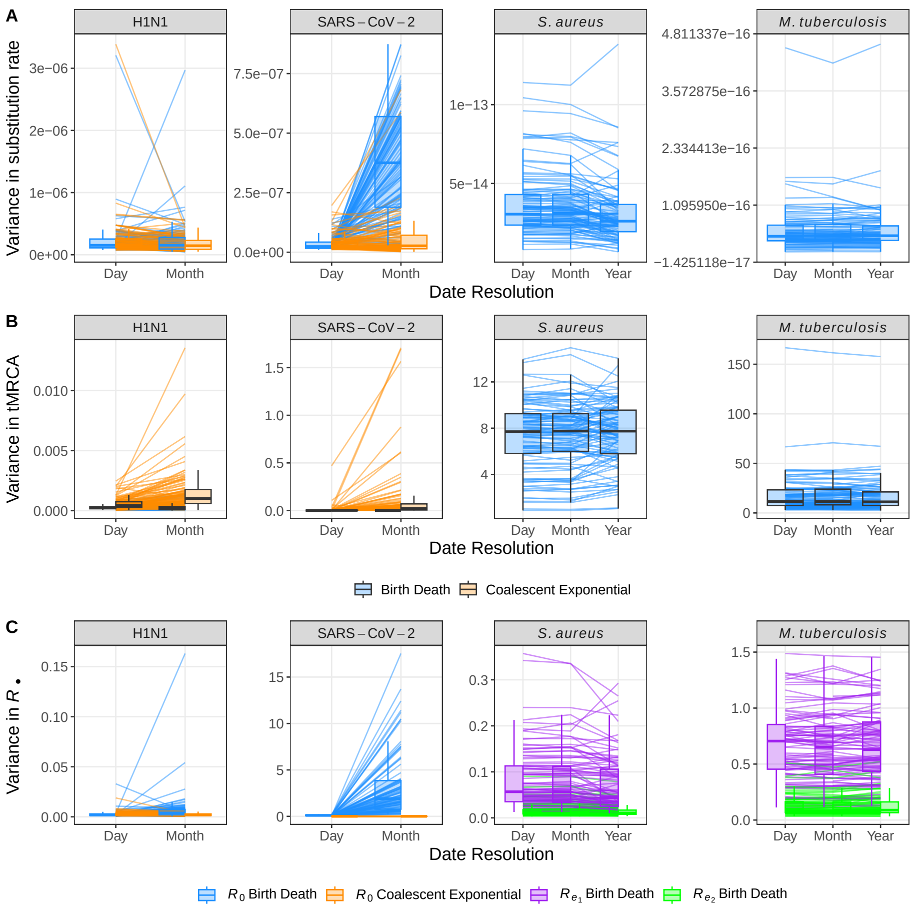

Mean posterior variance for parameters of interest for each replicate of simulated data varying across date resolution. Individual lines track mean posterior variance for each simulated dataset and boxplots are given to summarise variance in each condition at each date resolution. Rows correspond to individual parameters, columns correspond to simulation conditions (underlying parameters matching each empirical dataset), and colour corresponds to tree prior or reproductive number interval. (**A**) Mean posterior variance in substitution rate across simulation scenarios. (**B**) Posterior variance in tMRCA, a measure of the age of the population driving the outbreak. (**C**) Posterior variance in reproductive number reproductive number.

Fig. B

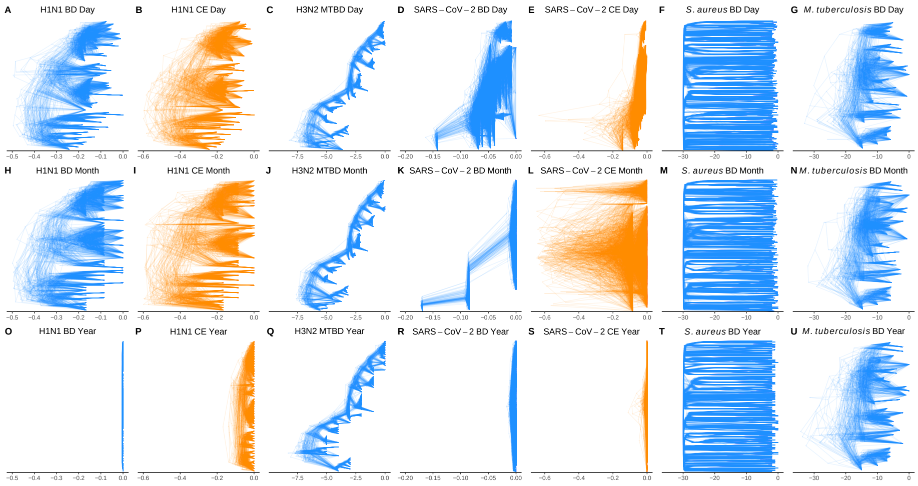

Desnsitrees (overlaid posterior trees) for empirical data with columns corresponding to pathogen under each combination of date resolution and tree prior. For the H1N1 and SARS-CoV-2 treatments, Year resolution causes trees to collapse to instantaneous bursts.

Fig. C

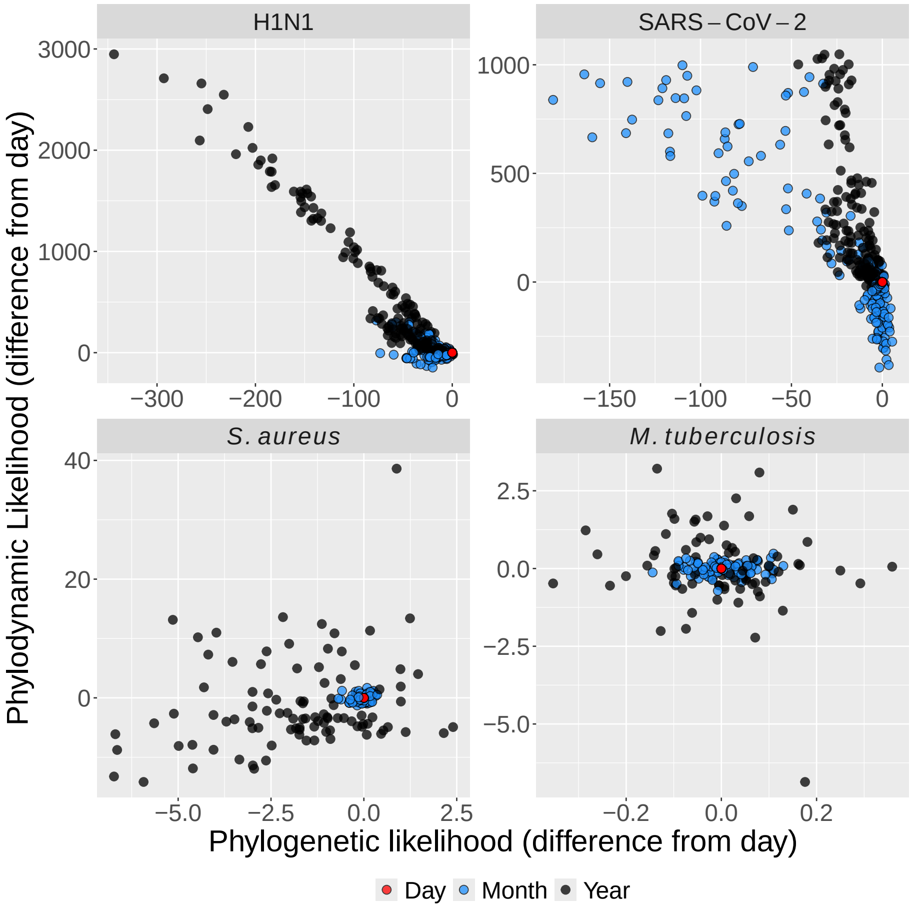

Adjusted phylodynamic likelihood against adjusted phylogenetic likelihood with panels corresponding to each simulation condition. Points correspond to mean posterior likelihood for each simulated dataset under each simulation condition. Colour corresponds to date resolution. Likelihoods are adjusted by subtracting the mean phylodynamic or phylogenetic likelihood at Day resolution from each the means under Month and year resolution. Resulting points therefore show the difference phylodynamic and phylogenetic likelihoods due to date-rounding with the point $(0, 0)$ representing likelihood at day resolution for each dataset. Month resolution generally results in smaller differences that Year resolution, suggesting coarser date resolution results in more perturbed likelihoods. There is also generally more error in phylodynamic likelihood than phylogenetic likelihood.

Fig. D

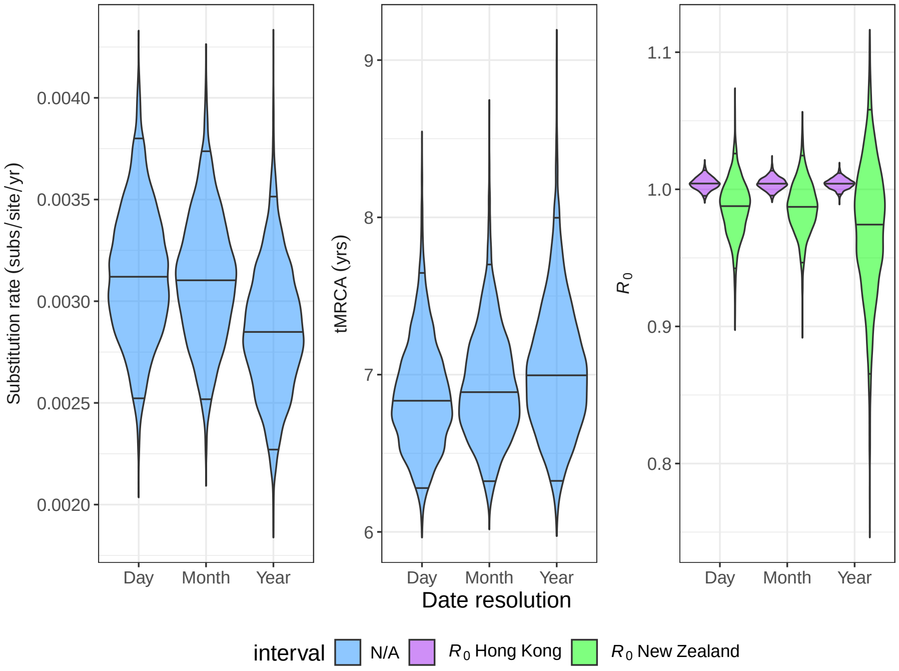

Posterior distributions for the substitution rate, tMRCA, and R_0 for the H3N2 dataset. The x-axis corresponds to the date resolution used in each re-analysis, and horizontal lines denote the median and 95% HPD bounds in each distribution. Overall, the H3N2 dataset is less sensitive to date rounding, but displays the same patterns of bias with decreasing date resolution as the comparable viral datasets of H1N1 and SARS-CoV-2 in the main text.

Fig. E

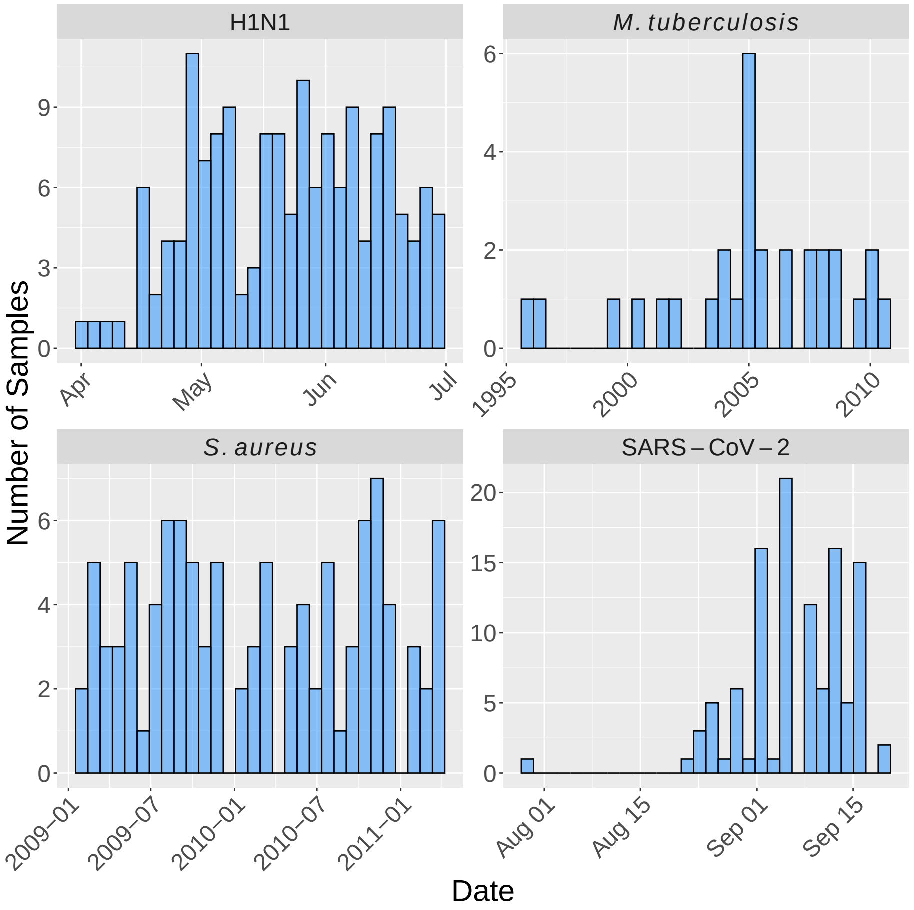

The number of samples over time for each empirical dataset. Date-rounding has the effect of moving each sampling within a month or year to the middle of that month or year (15th of the month or June 15th of the year).

Table. A

Mean posterior estimates of substitution rate and tMRCA for empirical data with 95% HPD in brackets. The lower table gives mean posterior estimates of $R_\bullet$ for empirical data with 95% HPD in brackets.

	Tree Prior	Resolution	Substitution Rate (subs/site/yr)	tMRCA
H1N1	BD	Day	3.76e-3 (2.7e-3, 4.9e-3)	4.04e-1 (3.4e-1, 5.1e-1)
H1N1	BD	Month	3.14e-3 (2.3e-3, 4.0e-3)	4.12e-1 (3.3e-1, 5.2e-1)
H1N1	CE	Day	3.01e-3 (2.0e-3, 4.1e-3)	4.86e-1 (3.8e-1, 6.5e-1)
H1N1	CE	Month	2.62e-3 (1.8e-3, 3.4e-3)	5.04e-1 (3.9e-1, 6.8e-1)
SARS-CoV-2	BD	Day	2.47e-4 (1.1e-4, 4.5e-4)	1.45e-1 (1.4e-1, 1.5e-1)
SARS-CoV-2	BD	Month	6.56e-4 (3.3e-4, 1.1e-3)	1.7e-1 (1.7e-1, 1.7e-1)
SARS-CoV-2	CE	Day	2.37e-4 (9.1e-5, 4.7e-4)	2.03e-1 (1.4e-1, 3.6e-1)
SARS-CoV-2	CE	Month	4.34e-5 (4.4e-6, 1.4e-4)	1.6 (2.9e-1, 5.9)
S. aureus	BD	Day	1e-5 (1e-5, 1e-5)	3e+01 (3e+01, 3e+01)
S. aureus	BD	Month	1e-5 (1e-5, 1e-5)	3e+01 (3e+01, 3e+01)
S. aureus	BD	Year	1e-5 (1e-5, 1e-5)	3e+01 (3e+01, 3e+01)
M. tuberculosis	BD	Day	1.02e-7 (6.5e-8, 1.4e-7)	2.17e+01 (1.7e+1, 3.2e+1)
M. tuberculosis	BD	Month	1.02e-7 (6.6e-8, 1.4e-7)	2.17e+01 (1.7e+1, 3.2e+1)
M. tuberculosis	BD	Year	9.86e-8 (6.2e-8, 1.4e-7)	2.25e+01 (1.8e+1, 3.4e+1)

	Tree Prior	Resolution	R_0	R_{e_1}	R_{e_2}
H1N1	BD	Day	1.07 (1.0, 1.1)	-	-
H1N1	BD	Month	1.12 (1.1, 1.2)	-	-
H1N1	CE	Day	1.13 (1.1, 1.2)	-	-
H1N1	CE	Month	1.12 (1.1, 1.2)	-	-
SARS-CoV-2	BD	Day	1.2 (9.3e-1, 1.6)	-	-
SARS-CoV-2	BD	Month	5.85 (3.7, 9.0)	-	-
SARS-CoV-2	CE	Day	1 (9.6e-1, 1.0)	-	-
SARS-CoV-2	CE	Month	1.01 (9.8e-1, 1.1)	-	-
S. aureus	BD	Day	-	1.57 (1.5, 1.7)	6.56e-1 (5.1e-1, 8.0e-1)
S. aureus	BD	Month	-	1.56 (1.5, 1.7)	6.78e-1 (5.4e-1, 8.3e-1)
S. aureus	BD	Year	-	1.73 (1.6, 1.8)	3.71e-1 (1.9e-1, 5.4e-1)
M. tuberculosis	BD	Day	-	2.77 (5.8e-1, 5.3)	1.4 (7.2e-1, 2.7)
M. tuberculosis	BD	Month	-	2.74 (5.7e-1, 5.0)	1.41 (7.4e-1, 2.7)
M. tuberculosis	BD	Year	-	2.66 (4.6e-1, 5.1)	1.53 (8.1e-1, 2.9)
