# Supplementary material for: How does date-rounding affect phylodynamic inference for public health?
Source: PLoS Comput Biol. 2025 Apr 11;21(4):e1012900. doi: 10.1371/journal.pcbi.1012900 (PMC11991728; doi:10.1371/journal.pcbi.1012900)
Supplement: Attachment — Submitted filename: response.pdf [file pcbi.1012900.s002.pdf]

19-12-2024

Dear Editor,

We would like to thank you and the two anonymous reviewers for your input on our manuscript. We have made changes in line with every suggestion, leading to what we feel is a stronger manuscript suited to the readership of *PLOS Computational Biology*.

The larger changes include analysis of a longer term H3N2 dataset reflecting 6 years of sampling, following the suggestion of reviewer 1 to consider a longer term viral dataset. We also include a supplementary figure showing that posterior uncertainty also increases with date rounding. These results have increased the nuance in the conclusions of this work, overall demonstrating that accurate sampling dates are critical to reduce error for emerging datasets reflecting short term sampling, with the resultant error decreasing for longer term sampling. We have emphasised this more clearly in the current submission, along with some editing to improve the overall clarity of the manuscript.

Below, we reproduce each comment and outline the action we have taken in blue.

Sincerely,

Leo A. Featherstone

Danielle Ingle

Wytamma Wirth

Sebastian Duchene

Editor

In addition to the reviewers' comments and suggestions, I would also appreciate if you could address the following points. First, I believe the use of term 'bias' is inconsistent. The error is described as "unpredictable in its direction and compounds with decreasing date-resolution", which sounds a lot like increasing variance to me. Bias, in contrast, would be that inferred dates are consistently younger when tip-date uncertainty is magnified [or inferred dates are older, but not both at the same time]. However, the directionality here appears to be highly model and organism dependent. Hence, it seems that tip-date uncertainty leads to greater variance and uncertainty, but not necessarily bias.

Response

Thank you for pointing out the lack of clarity in our reference to bias and error. It is indeed true that the bias we refer to from the quoted part of the abstract varies in direction in a way that is model and organism dependent. However, it is consistent within each combination of organism, model, and temporal resolution. Due to this, we feel that bias is a more appropriate term than error. We also have now included an analysis of the change in posterior variance for each estimate in the supplementary material (Fig S1), showing that posterior variance increases with reduced date resolution in a broadly similar pattern to the bias in estimates. To make this distinction clearer, we have included better distinction of each term in the abstract, introduction, and results as follows:

L9-14:

"*Here, we introduce a practical guideline on when date-rounding biases the inference of epidemiologically important parameters across a diverse range of empirical and simulated datasets. We show that the direction of bias varies for different parameters, datasets, and tree priors, while compounding with lower date-resolution and higher substitution rates. We also find that bias decreases for datasets with longer sampling intervals, implying that our guideline is most applicable to emerging datasets*"

L106-110:

"*Throughout this manuscript, we refer to bias where we recover error in estimated parameters with a consistent direction among replicates in our simulation study (consistently over- or underestimating). We do not chiefly consider the variance in posterior distributions of estimated parameters, but discuss this point in the results.*"

L293-295:

"*The posterior variance for each estimated parameter also tended to increase with decreasing date resolution, demonstrating that poorer date resolution increases the uncertainty as well as accuracy of parameter estimates (Figure S1).*"

Second, why does the Discussion directly name two pathogen nucleotide repositories (GISAID and Pathoplexus) and make no mention of other, larger governmental databases like GenBank, where GISAID and Pathoplexus currently import many sequences from?
Response
We have removed mention of specific repositories in this section to avoid unfairly neglecting any.

L467468: "*We urge others uploading data to repositories to include dates to the day where possible.*"

REVIEWER 1

Phylodynamic inference uses temporal information in the form of the sampling dates of the sequences to generate time-calibrated phylogenies from which tMRCAs, evolutionary substitution rates, and epidemiological parameters can be estimated. However, sampling month and day information are not always available, so strategies like rounding the sampling dates to the day or month are commonly used. Featherstone et al. present a comprehensive study of date-rounding's effect on the inference of important epidemiological and evolutionary parameters in a phylodynamic analysis, using both simulated and empirical datasets of viral and bacterial pathogens with varying evolutionary rates and sampling windows. The negative effects of rounding were more prominent for fast-evolving RNA viruses. The authors also argue that the bias introduced by date-rounding can be reduced (although not completely removed) when the unit of time used for the rounding (i.e., day or month) is shorter than the average time to observe a substitution in the sequences. Furthermore, they discuss some strategies to facilitate sharing complete sampling dates while protecting patient confidentiality.
The manuscript is well-structured and easy to read, the methods are clearly explained, and the results are discussed in detail. This study tackles one of the major issues in Phylodynamics, while also using relevant pathogens for the analyses. I have only some minor comments for this manuscript:

Why wasn't the coalescent prior used for the bacterial pathogens? Adding the reason would make the methods section clearer.
Response
We did not fit the coalescent exponential to the bacterial data because they reflect transmission beyond the exponential phase. In more practical terms, we are not aware of a segmented coalescent exponential that has been applied before. In fact, the lead

author spent some time trying to do this, but there is limited support for such modifications under the coalescent in BEAST.

We have clarified this in L187-189:
"*We did not fit the coalescent exponential to the bacterial datasets because they capture transmission beyond the exponential phase, which would therefore result in model misspecification.*"

I understand that it is recommended to avoid improper priors in a Bayesian phylogenetic analysis. What is the advantage of using an improper prior like 1/X over a Uniform with a very high upper bound (let's say 100 million for the population size)?
Response
The advantage of the 1/x over the uniform is that it is the Jeffrey's prior and maximally uninformative (https://doi.org/10.1093/genetics/161.3.1307), which is preferable given the goals of the simulation study. In addition, the uniform prior on population size does not lead to a uniform prior in root height (see Fig S5 here: https://doi.org/10.1371/journal.pcbi.1012371) and would introduce an extraneous source of noise when interpreting the tMRCA. Were we to do marginal likelihood, we would need to use another prior, but this is out of scope here. We have added clarification around this prior choice in L206-209:

"*…We also placed an improper prior (1/x) on the effective population size, which is the maximally uninformative Jeffrey's prior for coalescent intervals (Drummond et al., 2002).*"

Line 200: delete every in "since every the target"
Response
Done

Line 203: could the authors add a brief explanation of the origin parameter? Does it represent the age of the outbreak mentioned in line 186?
Response
We have added this explanation in L219-221:
"In this case, the origin parameter corresponds to the length of the root branch. In the results we still report the age of the outbreak as the tMRCA for consistency with the other datasets"

Line 230: replace "birth-tree" by "birth-death tree"
Response
Done

Line 271 remove duplicated words: At the year resolution there is there is
Response
Done

In figure 3, panel B, the coalescent results are missing for SARS-CoV-2. Was this done on purpose? If yes, add the justification on the figure legend.
Response
Thank you for spotting this. We indeed did omit the coalescent results for the coalescent because the posterior was effectively flat over 0 to 26 years, obscuring the other distributions. We now point this out in the figure 3 legend and provide a link to the unfiltered figure online:

"*Posterior tMRCA for SARS-CoV-2 data under the coalescent exponential are omitted because they are effectively flat over zero to twenty-plus years and obscure the other distributions. The un-filtered figure is available online.*"

REVIEWER 2
The manuscript entitled "How does date-rounding affect phylodynamic inference for public health?" presents an elegant analysis with a clear public health application. The authors investigate whether rounding of the sampling date introduces bias in phylodynamic inference, along with the magnitude and directionality of this bias based on several parameters. They also present a solution of how to avoid the rounding while preserving patient's confidentiality. Overall, the article is well written and presented and I recommend it for publication. A few suggestions/questions that could help improve the manuscript include:
1. Is there a possibility to include an example of a viral rapidly evolving pathogen sampled over a longer period of time (a few years or decades)? I wonder if the same "clustering in time" issue will be a problem when there are more clusters that are distributed over time (i.e. samples collected over 48 months rather than 9). As we are moving into year 5 of COVID and year 40+ with HIV, it would be interesting to consider how the dates resolution affect phylodynamic inference of viral infections of public health concern sampled over a longer period of time.
Response
We thank the reviewer for pointing this out and acknowledge that we did search for longer term datasets when sourcing data for the paper. We were unable to find a longer term HIV or SAR-CoV-2 dataset that was freely available and had complete information. Instead, we have now identified a H3N2 influenza dataset sampled over 6 years between Hong Kong and New Zealand. We have included a supplementary analysis of this dataset, showing that bias due to date resolution is lesser for longer term sampling. This has added depth to our results, by highlighting the severity of bias due to date

rounding for short-term datasets. Moreover, the H3N2 dataset is ideal in requiring a multi-type birth-death analysis, allowing us to also consider the effects of date rounding under a more complicated model. We have updated the text in the introduction, methods, results, and discussion following the inclusion of these data

Introduction L128-129:

"*We also included a supplementary H3N2 influenza empirical dataset to illustrate the effects of date rounding on longer-term viral datasets.*"

Methods L235-247:

"*We included a supplementary viral dataset to observe the effects of date rounding for datasets from longer term sampling. Using the multi type birth-death model, we analysed a datasets of 60 H3N2 influenza samples taken from 2000 to 2005 in Hong Kong and New Zealand, with demes corresponding to each location (Kuhnert et al., 2016). The data are a subset of the data originally used in Vaughan et al. (2014), and is also available from the structured birth-death 'Taming the BEAST' tutorial (Barido-Sottani et al., 2017). We placed a Lognormal(μ = 0, σ = 1) prior on R0 for both demes and fixed the becoming un-infectious rate at δ = 71, corresponding to a roughly 5-day duration of infection. We also placed Lognormal(μ = 0.001, σ = 1.25) on the substitution rate and sampling probability (p). The latter was fixed to zero at above the height of 5.7 years: that is, shortly before the oldest sample in all date treatments*"

Results L403-415:

"*The H3N2 data were more robust to date rounding than the H1N1 and SARS-CoV-24 datasets, which were sampled over shorter time frames (Figure S3). Posterior R0 for Hong Kong, from which the majority of samples originate, remains nearly identical across date resolutions (mean posterior values of 1.00 for each date resolution). Posterior R0 for the New Zealand deme also remains near-identical at day and month resolution, before increasing in uncertainty at year-resolution (mean values of 0.99, 0.99, and 0.97 respectively). The mean posterior substitution rate decreases at year resolution ($3.13x10^{-3}$, $3.11x10^{-3}$, $2.86x10^{-3}$), while the tMRCA increases in step with each reduction in date resolution from day, to month, to year (6.87, 6.93, 7.04 The relative stability of posterior estimates across date resolutions for the H3N2 dataset differs from the bias induced in the H1N1 and SARS-CoV-2 datasets, supporting the notion that shorter sampling spans exacerbates bias due to date-rounding.*"

Discussion L487-515:

“*Longer sampling intervals also reduce bias due to date-rounding. This is demonstrated by supplementary analysis of the H3N2 data, spanning roughly 6 years. Posterior estimates of R0, tMRCA, and substitution rate vary much less with lower date-resolution than for the other H1N1 and SARS-Cov-2 datasets sampled over three and two months respectively. In turn, the H1N1 dataset also demonstrates lesser bias than the SARS-CoV-2 dataset. This is consistent with previous results for ancient DNA data showing that date-rounding has negligible effects for timescales of millennia or longer, where the sampling span covers the average substitution interval many times (Molak et al., 2013). The mitigating effect of sampling duration implies that the hypothesis relating bias to average substitution time and date resolution is of practical significance for emerging datasets sampled over shorter time frames. Conversely, datasets reflecting longer-term sampling are more likely to be robust to date-rounding.*

Determining the sampling duration after which inference is sufficiently robust to date-rounding requires case-by-case consideration. The answer will reflect multiple factors including of the pathogen’s substitution rate, the number and temporal-distribution of samples, and aspects of inference including the choice of model and priors. For example, although roughly six years of sampling largely eliminated bias at the month resolution for the H3N2 data, a longer time frame would be needed for a bacterial dataset to span an equivalently informative amount of substitutions. Moreover, we note that longer sampling duration improves bias by including more internal branches that drive a signal for lower rates of substitution and transmission, counteracting the opposite signal local to each cluster of sampling times. That is, longer sampling durations introduce a balancing source of error, rather than directly accounting for the inaccuracy in sampling times. Is is therefore possible that extremely long sampling durations in combination with date rounding could exacerbate the opposite bias, inferring erroneously low substitution and transmission rates. Given this potential confounding factor, we suggest that the best solution is prevention wherein the most precise dates that balance patient confidentiality and phylodynamic accuracy are prioritized”

2. It is not obvious to me why the authors insist on including the coalescent tree prior for SARS-CoV-2 dataset even though it violates the sampling proportion assumption (as they highlight in the text). Since this tree prior is already not included for the bacterial infections, I would consider removing.

Response

We can appreciate that the coalescent exponential (CE) offers little for the SARS-CoV-2 datasets per se, but we have opted to retain it as it offers a useful comparison to the CE for the H1N1 data and demonstrates that the effects of date rounding emerge distinctly

from those of a poorly fitting tree prior, in the sense of bias increasing with decreasing date resolution. We highlight this more clearly in the methods L227-233:

"*Note that the coalescent exponential is not a natural choice of tree prior for the SARS-CoV-2 data because of its very high sequencing proportion (Stadler et al., 2015). We nevertheless include it for the SARS-CoV-2 data to provide a comparison to the coalescent exponential for the H1N1 data, and an example of how data-rounding may exacerbate error due to poorly fitting models. The model's poor fit is reflected later in the results.*"

And later in the discussion L523-525:

"These patterns emerged even for the coalescent exponential fit to the SARS-CoV-2 empirical and simulated data, for which it is a poorly fitting tree prior. This highlights that date rounding has a pervasive effect distinct from the effects of a poorly fitting tree prior."

3. Also, I understand that there is no effective "true value" for the empirical datasets, but maybe a range of "plausible values" based on the literature could be helpful for figure 3.

Response

We are reluctant to provide reference values for these plots because of the results because the tMRCA and reproductive number will naturally vary with the specifics of the outbreak and host population. However, we can understand the need for a reference point, so we have included median and 95% HPD bars to help aid a comparison between each posterior in the figure. We feel this is a good compromise, since the paper is at its core focussing on the difference in posterior estimates are date resolution changes. We have also updated the figure legend in accordance.

4. I suggest that the last paragraph in the Introduction to be moved to the Methods as it refers to the study setting and design.

Response

The final paragraph of the Introduction, does not refer to the study setting and design, other than by mentioning that we conducted empirical analyses and a simulation study which are introduced earlier in the introduction. We opted not to move this paragraph due to this.

5. Line 123-124 – did you mean "rounded to weeks"? If not, then unclear why Figure 1 refers to weeks and not days.

Response
We have updated figure 1 to emphasise rounding to the day more clearly.

6. A few questions about the simulation setup that are not clear from the text: please explain how you define the proportion of sequenced cases in your simulations to meet the empirical datasets. Also, what did you consider to be the outbreak duration in your simulation?
Response

We define the sequencing proportion in each simulation setting by matching it to the value used or employed in the originating source. We now clarify this in L154-158:

"*We matched the values of each parameter to those in the originating literature (Table 1). We fixed the sequencing proportion in the H1N1 simulations by dividing the sample size (n = 161) and the cumulative number of North American cases over the empirical data's sampling interval, resulting in the order of 1% (Jhung et al., 2011).*"

We also indicate that the outbreak duration corresponds to the parameter T in L153 as well as in the caption for Table 1:

"*Simulations consisted of four parameter settings corresponding to each empirical dataset (Table 1), with replicates of each. All parameter sets include a proportion of sequenced cases (p), outbreak duration (T).*"

Please describe the tree priors at the first time you refer to them (line 160).
Response
We have described and referenced each as suggested in L175-176.

Figure 2 – order legend in the same order as colors appear in the figure.
Response
Done for both Figure 2 and Figure 3.

Line 271 and 395 typos.
Response
We corrected L271, however it is unclear what the reviewer was pointing to in L395.

OTHER CHANGES

We also wish to note that we found a small bug in the rounding of H1N1 empirical dates (rounding to the 1st of each interval rather than the halfway point - i.e. 01/01 vs 01/15).

We have remedied this in this submission, and adjusted our wording around Figure 4 regarding likelihood divergence, which presents a result now more consistent with the H3N2 dataset. This adds nuance, but does not change the conclusions or results of the paper qualitatively.

L359-370:
"*Phylodynamic and phylogenetic likelihoods also appear to diverge when date resolution is less than the average substitution time (Figure 4). The SARS-CoV-2 data show a clear separation in likelihoods from day to month resolution for both tree priors. Likelihoods also separate at year resolution for the S. aureus data and remain nearly identical for M. tuberculosis. However, likelihoods overlap considerably at day and month resolution for H1N1 under both tree priors, which differs from expectation. This is probably due to longer sampling duration, compared to the SARS-CoV-2 data, lessening the effects of data rounding. Together, these movements in likelihood distributions support the hypothesis that date resolution less than the average substitution time leads to bias, with this effect reducing for longer sampling duration*"
